# Assessment of sexual function and quality of life of patients with spinal cord injury

**DOI:** 10.1192/j.eurpsy.2023.2326

**Published:** 2023-07-19

**Authors:** A. Loghmari, A. Mtiraoui, K. Ben Ahmed, K. Bouassida, M. Ben Othmen, G. Tlili, E. Acacha, W. Ben Abdallah, M. Kahloul, W. Hmida, M. Jaidane

**Affiliations:** 1 Urology, Sahloul teachin Hospital Sousse; 2 Psychiatry, Farhat hached Hospital, Sousse; 3 Urology, Sidibouzid Hospital, Sidibouzid; 4 Anesthesiology department, Sahloul teachin Hospital Sousse, Sousse, Tunisia

## Abstract

**Introduction:**

Spinal cord injuries are known to have physical damage. They are often accompanied by urinary and fecal incontinence, disorders of tenderness, neuropathic pain and motor deficits. Therefore they have a serious impact on physical, mental and social health.

**Objectives:**

The objective of our work was to assess sexual function and quality of life in men with spinal cord injuries.

**Methods:**

This is a cross-sectional study enrolling patients with spinal cord injuries followed at the physical medicine consultation and/or urology department of Sahloul teaching Hospital during the period from January 2016 to January 2018.

**Results:**

This study enrolled 21 patients. Mean age was 45.62 ±15.79 years. Thirteen patients were married, and nine had a primary school level of education. Thirteen patients worked as building workers. The cause of the spinal cord injury was traffic accident in 12 cases. The overall IIEF 15 was 15.57±7.46. Thirteen patients had erectile dysfunction which was rated severe in five patients. The average MSQ was 27.52 ± 26.32 with 10 patients very dissatisfied. The overall SF-36 score was 31.71± 26.16. We found a statistical correlation between quality of life impairment and sexuality impairment in almost all dimensions and especially impairment of physical activity (r²=+0.783 p<0.001) and impairment of perceived health.

**Conclusions:**

The impairment of sexual function is a serious health problem in patients with spinal cord injury. It has a serious impact in the quality of life that justify specific interventions.

**Disclosure of Interest:**

None Declared

